# Evaluation of emergency drills effectiveness by center of disease prevention and control staff in Heilongjiang Province, China: an empirical study using the logistic-ISM model

**DOI:** 10.3389/fpubh.2024.1305426

**Published:** 2024-02-27

**Authors:** Ruiqian Zhuge, Adelina Ruzieva, Na Chang, Xing Wang, Xinye Qi, Qunkai Wang, Yuxuan Wang, Zheng Kang, Jingjing Liu, Qunhong Wu

**Affiliations:** ^1^Department of Social Medicine, Health Management College, Harbin Medical University, Harbin, China; ^2^School of Public Health, Anhui University of Science and Technology, Huainan, Anhui, China; ^3^Key Laboratory of Industrial Dust Prevention and Control, Occupational Safety and Health, Ministry of Education, Anhui University of Science and Technology, Huainan, Anhui, China; ^4^Anhui Institute of Occupational Safety and Health, Anhui University of Science and Technology, Huainan, Anhui, China; ^5^Joint Research Center of Occupational Medicine and Health, Institute of Grand Health, Hefei Comprehensive National Science Center, Anhui University of Science and Technology, Huainan, Anhui, China

**Keywords:** emergency drills, effectiveness evaluation, logistic-ISM model, influencing factors, center of disease prevention and control staff

## Abstract

**Introduction:**

Emergency drills are critical practices that can improve the preparedness for crisis situations. This study aims to comprehend the evaluation of emergency drill effectiveness by the staff at the Centers for Disease Control and Prevention (CDC) in Heilongjiang Province, China. It identifies potential factors that could influence the personnel’s appraisal of outcomes throughout the emergency drill procedure.

**Methods:**

A cross-sectional survey was conducted among public health professionals from various CDCs in Heilongjiang, a northeastern Chinese province. The binary logistic regression analysis identified the factors associated with the CDC staff’s assessment of emergency drill efficacy, while the Interpretative Structural Modeling (ISM) elucidated the hierarchical structure among the influencing factors.

**Results:**

53.3% (95% CI = 50.6–55.4) of participants perceived the emergency drills’ effectiveness as low. Binary logistic regression analysis revealed that the following adverse factors associated with the emergency drills increased the risk of a lower evaluation: lack of equipment and poor facilities (OR = 2.324, 95% CI = 1.884–2.867), poor training quality (OR = 1.765, 95% CI = 1.445–2.115), low leadership focus (OR = 1.585, 95% CI = 1.275–1.971), insufficient training frequency (OR = 1.539, 95% CI = 1.258–1.882), low skill in designing emergency drill plans (OR = 1.494, 95% CI = 1.180–1.890), lack of funding (OR = 1.407, 95% CI = 1.111–1.781), and poor coordination between departments (OR = 1.335, 95% CI = 1.085–1.641). The ISM revealed the hierarchical relationship of the influential factors, which were classified into three levels: Surface, Middle and Bottom. The Surface Level factors were training frequency, training quality, leaders’ focus, and inter-departmental coordination. The Middle Level factors were equipment availability and skill in designing emergency drill plans. The Bottom Level factor was funding guarantee.

**Discussion:**

This survey revealed that over half of the CDC staff rated the effectiveness of public health emergency drills as low. The Logistic-ISM Model results indicated that the evaluation of drill effectiveness was negatively influenced by insufficient facility and equipment support, financial constraints, lack of departmental coordination, and inadequate leadership attention. Among these factors, funding guarantee was the most fundamental one. Therefore, this calls for strategic decisions to increase funding for equipment, leadership training support, and effective emergency coordination.

## 1 Introduction

Many disasters and emergencies are unpredictable and need to be tested through practical simulations to enhance the preparedness of the personnel involved. Emergency drills are useful tools for this purpose (1–3). Emergency drills allow departments and individual practitioners to evaluate their ability to respond quickly and effectively to real-world crises (4). Since these drills often occur without prior notice, responders must show rapid and accurate responses (5, 6). Simulated emergency drills are essential components of any robust emergency preparedness framework (7). They aim to be part of a continuous cycle that includes planning, training, practicing, identifying weaknesses, improving areas, and implementing corrective actions.

According to the “Technical Guidelines for Health Emergency Drills” published by the Chinese Center for Disease Control and Prevention in 2013, emergency drills can be understood as a simulated training and experiential learning process for responding to sudden public health events (8). “How to deal with accidents, how to conduct drills,” just like “how to fight, how to train soldiers.” In summary, emergency drills are by no means a simple repetition of past emergency practices. They emphasize the exploration of standardized emergency response or the simulation of “worst-case scenarios” (9). Furthermore, drills are training, which emphasizes “scenario-driven, role-playing, teaching interaction, and joint evaluation (10).”

After the devastating SARS outbreak in 2003 and the Wenchuan earthquake in 2008, the Chinese Central Government shifted its focus to establishing an integrated national emergency response system that can anticipate, assess, mitigate, and manage potential disasters whenever possible (11). Amid the escalating COVID-19 crisis in 2019, the Central Government gave more priority to the role of CDCs in protecting public health and intensifying health emergency drills. However, for emergency drill projects, people’s evaluation of their effectiveness is often unsatisfactory.

Studies have shown that the design, implementation and organization of drills can affect the testing and improvement of emergency drill procedures, which in turn affect the evaluation of the effectiveness of emergency drill (4, 12–14). Enough funding, equipment, and logistical support are needed to make the simulations mimic life-threatening situations and prepare for actual events (15). For example, a four-day emergency exercise in New South Wales, Australia, simulated a new strain of the flu virus, with enough equipment backup and technical support and keeping it authentic through strict monitoring. The responders did well when H1N1 hit just 8 months later (16). This resulted in efficient emergency response efforts and less economic damage. However, after the SARS outbreak in China, the main form of training for two decades was short lectures that lacked specificity and novelty (17). The participants were diverse and not very engaged, making it hard for the training to meet their needs. Some of the other limitations are lack of funding, unclear or vague guidelines, lack of interest from senior leaders, coordination problems among departments, lack of institutional security frameworks, no direct link to employee evaluations, and low motivation among workers (18–22). All these factors will have a negative impact on people’s evaluation of emergency drills. While emergency drills are becoming more popular worldwide because they help gain practical skills quickly, there is not much research on their use, suitability, and efficiency in China, even though they are getting more recognition.

To evaluate the relevant factors for the effectiveness evaluation of emergency drills, the Logistic regression model can be used. However, the Logistic regression model cannot reveal the hierarchical relationship among these relevant factors (23, 24). To do this, the ISM method can be further used to analyze the significant factors in the Logistic regression model, and thus identify the surface, intermediate and bottom factors. ISM is a technique for analyzing complex problems by creating structural models, developed by Professor Warfield in 1973 (23). It uses a matrix model to decompose the complex relationship among relevant factors into a clear multi-level structure through a grouping and ranking process (24).

In summary, this study aimed to understand how CDC personnel in Heilongjiang Province evaluated the effectiveness of emergency drills based on their feedback and insights, using the Logistic-Ism method, to identify the obstacles and potential factors that influenced their appraisal of outcomes, and to display the hierarchical relationship between these factors. The results of this study not only help to gain a deeper understanding of the current situation of the effectiveness evaluation of emergency drill projects by the Heilongjiang CDC staff, but also provide an important basis for optimizing the ways to improve the effectiveness of emergency drills.

## 2 Methods

### 2.1 Data collection

This study conducted a cross-sectional anonymous survey of disease prevention and control practitioners at the municipal, district, and county levels in Heilongjiang, China. We used a multistage stratified cluster sampling method to select the participants. We randomly sampled seven administrative regions out of the 13 in Heilongjiang province in the first stage. In the second stage, we performed cluster sampling of the 40 municipal, district, and county-level disease prevention and control institutions within these seven regions. We distributed paper questionnaires to all staff members in each institution. The inclusion criteria for this study were as follows: (1) voluntary participation, (2) signing an informed consent form, (3) having participated or known someone who had participated in at least one emergency drill project, and (4) providing personal assurance of the accuracy of the information. The exclusion criteria were as follows: (1) missing key items in the questionnaire, (2) contradictory answers, and (3) too short or too long response time.

### 2.2 Questionnaire design

**Figure 1:**
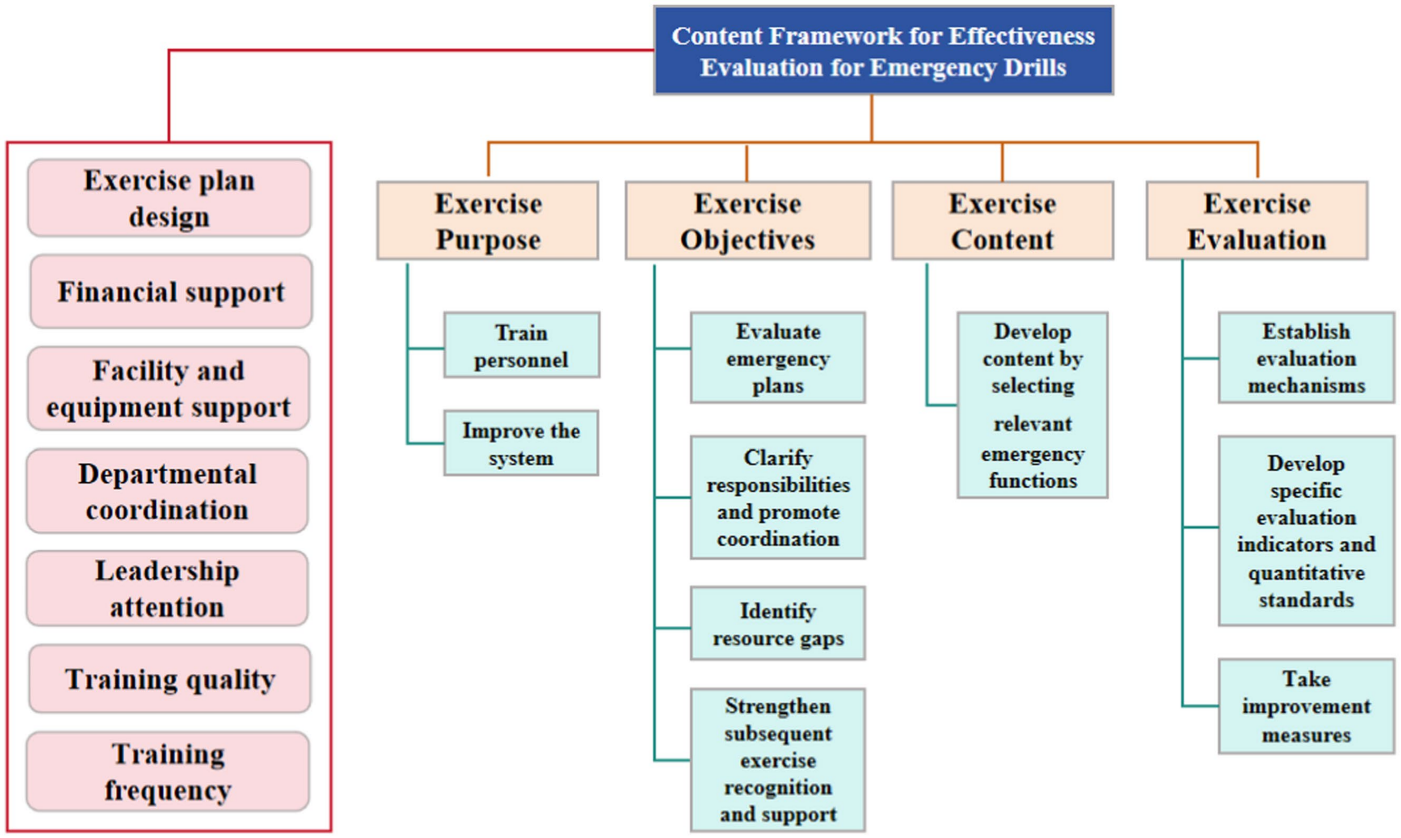
Reference content framework for evaluation of the effectiveness of emergency drills.

To evaluate the effectiveness of emergency drills, this survey questionnaire was designed based on the ‘Technical Guide for Health Emergency Drills’ issued by the Chinese Center for Disease Control and Prevention (8). It enumerated several evaluation criteria for the participants’ reference. The participants were encouraged to consider factors such as financial support, facilities and equipment, departmental coordination, and leadership attention to ultimately provide an overall assessment score. The content for validity assessment could be divided into the following aspects:

1. Exercise purpose: Although emergency drills can achieve many specific objectives, they can be generally summarized into two aspects: First, whether the staff were trained, enabling the participants to access their respective emergency functions and roles, and thus gain more skills and experience. Second, whether the system was improved, by refining the health emergency plan through the drill, and further promoting the comprehensive improvement of the health emergency management system.
2. Exercise objectives: First, whether the existing health emergency plans, implementation schemes, and operating procedures were tested and evaluated, and whether the shortcomings were revealed. Second, whether the responsibilities of each department and organization were clarified, and whether the coordination and communication among them were strengthened. Third, whether the personnel with corresponding emergency functions and roles were trained, and whether their abilities and levels were improved. Fourth, whether the lack of resources for health emergency work was revealed. Fifth, whether the recognition and support for the emergency drill planning were enhanced in the follow-up.
3. Exercise content: Whether the relevant emergency functions were selected for content development according to the purpose and objectives of the drill, such as monitoring and early warning, risk assessment, field epidemiological investigation, field rapid testing, laboratory testing and identification, etc.
4. Exercise evaluation: whether there is a scientific and rigorous evaluation mechanism, specific evaluation indicators and quantitative standards, and whether corresponding improvement measures have been taken after the drill (Figure 1).

### 2.3 Variable

#### 2.3.1 Outcome variable

The outcome measure was the overall evaluation of the effectiveness of emergency drills by CDC personnel. This was assessed by a single item, “How do you rate the effectiveness of the emergency drill program conducted by your unit?” The results were graded on a five-point Likert scale (ranging from 1 = very poor to 5 = very good). These scores were divided into two categories for logistic regression modeling: “high effectiveness” (scores above the average of 3.16) and “low effectiveness” (scores equal or below the average of 3.16).

#### 2.3.2 Explanatory variable

The explanatory variables were divided into several categories according to previous studies, such as demographics (gender, age, education, work tenure), previous training experience (frequency and quality of training sessions), components of emergency drills (funding, personnel, site, equipment, security, leadership, and coordination), types of exercises conducted in the past 5 years (tabletop, operation-based, functional, and comprehensive), and emergency drill disposal skills (plan designing, script writing, form designing, and needs assessment).

Age was divided into four groups, namely, less than or equal to 30 years, 30–39 years, 40–49 years, and greater than or equal to 50 years; work tenure was also divided into four categories, namely, 0–9 years, 10–19 years, 20–29 years, and greater than or equal to 30 years. The number of training sessions was binary (“none” or “≥1”), the quality of training was either “low quality “or “high quality “, the components of emergency drills were either “yes” or “no,” and the answers for emergency drill disposal skills were rated on a 5-point scale, ranging from 1 (very poor) to 5 (very good). In order to apply the binary logistic regression method, the answers were finally coded as two-dimensional, including bad (0) and good (1). 1, 2, and 3 were classified as bad, and 4 and 5 were classified as good.

Among the four types of emergency drills used in the past, a tabletop drill is a discussion of different measures based on a hypothetical scenario, an operation-based drill is a practice of actual combat in a real-life setting, a functional drill is a drill based on an emergency response function or some of its actions, a comprehensive drill is a drill that covers all or most of the emergency response functions in an emergency plan.

### 2.4 Statistical analysis

This study applied two methods to analyze the survey data: binary logistic regression, which examined the effect of individual predictors on the main outcome variable, and Interpretative Structural Modeling (ISM), which explored the interconnections and hierarchies of factors. The data were organized and analyzed using SPSS V.19.0 and MATLAB V.9.0. First, univariate descriptive statistics were obtained for all variables considered in the study. Then, Pearson’s *χ*^2^ was used to test the association between all variables and self-assessment of the effectiveness of emergency drills, with a two-tailed value of *p* < 0.05 indicating statistical significance. Factors that were significant in the *χ*^2^ test were included in the regression analysis. Then, the significant factors were further screened by using binary logistic regression analysis, and the hierarchical structure among them was revealed by using ISM method.

The specific steps of ISM analysis are as follows:

Step 1: Judge the logical relationship between the influencing factors and construct the adjacency matrix R.

The adjacency matrix is a way of representing the matrix of relationships between factors. It provides a clear description of the direct relationship between factors. In this study, $S_{0}$ is used to refer to the selection of the effectiveness of emergency drills for CDC responders. On the other hand, $S_{i}$ (=1,2,…,k) is used to denote the k significant factors that affect the evaluation of the effectiveness of emergency drills. The adjacency matrix R is constructed based on the logical relationship between the factors, which is defined as follows:


$$R_{ij}=\{\begin{array}{c} 1,\;S_{i}\;\mathrm{is}\;\mathrm{related}\;to\;S_{j}\;\;\\ 0,\;S_{i}\;\mathrm{is}\;\;\mathrm{not}\;\mathrm{related}\;to\;S_{j}\; \end{array}\;\mathrm{Among them},i=0,1,2\ldots ,k;j=0,1,2\ldots ,k\text{.}$$

The adjacency matrix $R_{ij}$ represents the binary direct relationship between the factors within the system through the form of a 0–1 matrix. Through extensive information gathering and consultation with experts, we can determine the direct relationship between factors that affect the evaluation of the effectiveness of emergency drills for CDC personnel. If $S_{i}$ directly affects $S_{j}\text{,}$ we assign a value of 1 to $R_{ij}$ . If not, we assign a value of 0. By doing so, we can generate the adjacency matrix, *R*.

Step 2: Based on the adjacency matrix R, create the reachable matrix M.


$$M={\left(R+I\right)}^{\lambda +1}={\left(R+I\right)}^{\lambda +1}\neq \ldots \ldots {\left(R+I\right)}^{2}\neq \left(R+I\right)\text{.}$$

The reachable matrix describes the relationship between factors’ reachability in the system. Where *I* is the unit matrix, 2 ≤ λ ≤ *k*, and the Boolean operator is used for the power operation of the matrix.

Step 3: Determine the hierarchical structure L between the factors.


$$L=\left\{S_{i}|\;R\left(S_{i}\right)\cap A\left(S_{i}\right)=Q\left(S_{i}\right)\right\}\left(i=0,1,2\ldots ,k\right)\text{.}$$

where $R\left(S_{i}\right)$ is the reachable set and $A\left(S_{i}\right)$ represents the prior set. After obtaining the elements contained in the highest layer *L*, the rows corresponding to the factors in *L* are rounded off from the original reachable matrix *M* to obtain the matrix $M_{2}$ , which is repeated to obtain $L_{2}$ , and so on the factors contained in all layers are obtained, and the hierarchical structure of the factors influencing the evaluation of the effectiveness of emergency drills is finally obtained.

### 2.5 Ethical considerations

This study adhered to the Declaration of Helsinki principles and received approval from the Institutional Review Board ethics committee at Harbin Medical University. The study subjects gave informed consent before participating. The information collected was anonymous and confidential to safeguard the privacy of the subjects.

## 3 Results

### 3.1 Characteristics of participants and self-rated effectiveness of emergency drills

The survey sample consisted of 1,859 subjects, of whom 59.1% were female. The majority of the participants (84%) had a Bachelor degree or higher, and 37.7% had more than 20 years of work experience. Most participants were in the age range of 30 to 49 years (Table 1).

**Table 1:** Characteristics of participants (*N* = 1859).

Variable		*N* (%)
Gender	Male	761 (40.9)
	Female	1,098 (59.1)
Age group	≤ 30 years old	238 (12.8)
	30–39 years old	558 (30.0)
	40–49 years old	773 (41.6)
	≥50 years old	290 (15.6)
Education background	Junior college and below	297 (16.0)
	College and above	1,562 (84.0)
Work tenure	0–9 years	666 (35.8)
	10–19 years	491 (26.4)
	20–29 years	521 (28.0)
	≥30 years	181 (9.7)

The training methods differed in frequency and type. Operations-based drills were the most common methodology reported by participants, accounting for 36% of the total training sessions. The next most common method was tabletop drills (31%), followed by function-oriented drills (20%) and comprehensive drills (13%). More than half (53.3, *95% CI* = 50.6 to 55.4) of the 1,859 participants rated the overall effectiveness of the emergency drills as low.

### 3.2 Chi-square test and binary logistic regression results

Using χ^2^ test we identified the statistically significant association between the perceived effectiveness of emergency drills and gender, educational background, training frequency, training quality, fund guarantee, equipment and facilities availability, leaders’ focus, inter-departmental coordination, tabletop drill exercises, operation-based drill exercises, and skills in emergency drill plan design, script writing, form design, and needs assessment (Table 2).

**Table 2:** Chi-square test results of all explanatory variables (*N* = 1859).

Variables		High effectiveness (*n* = 869) *n* (%)	Low effectiveness (*n* = 990) *n* (%)	*Χ^2^*	*P*
Gender	Male	393 (45.2)	368 (37.2)	12.4	0.000
	Female	476 (54.8)	622 (62.8)		
Age group	≤ 30 years old	114 (13.1)	124 (12.5)	0.3	0.959
	30–39 years old	261 (30.0)	297 (30.0)		
	40–49 years old	362 (41.7)	411 (41.5)		
	≥ 50 years old	132 (15.2)	158 (16.0)		
Education background	Junior college and below	118 (13.6)	179 (18.1)	7.0	0.008
	College and above	751 (86.4)	811 (81.9)		
Work tenure	0–9 years	325 (37.4)	341 (34.4)	2.4	0.500
	10–19 years	228 (26.2)	263 (26.6)		
	20–29 years	231 (26.6)	290 (29.3)		
	≥ 30 years	85 (9.8)	96 (9.7)		
Training frequency	None	396 (45.6)	337 (34.0)	25.8	0.000
	≥ 1	473 (54.4)	653 (66.0)		
Training quality	Low quality	442 (50.9)	379 (38.3)	29.7	0.000
	High quality	427 (49.1)	611 (61.7)		
Fund guarantee	Yes	612 (70.4)	790 (79.8)	21.9	0.000
	No	257 (29.6)	200 (20.2)		
Personnel guarantee	Yes	452 (52.0)	484 (48.9)	1.8	0.193
	No	417 (48.0)	506 (51.1)		
Drill site guarantee	Yes	338 (38.9)	351 (35.5)	2.4	0.125
	No	531 (61.1)	639 (64.5)		
Equipment and facilities guarantee	Yes	412 (47.4)	683 (69.0)	89.0	0.000
	No	457 (52.6)	307 (31.0)		
Security guarantee	Yes	254 (29.2)	285 (28.8)	0.0	0.834
	No	615 (70.8)	705 (71.2)		
Leaders’ focus	Yes	474 (54.5)	647 (65.4)	22.6	0.000
	No	395 (45.5)	343 (34.6)		
Inter-departmental coordination	Yes	373 (42.9)	533 (53.8)	22.1	0.000
	No	496 (57.1)	457 (46.2)		
Tabletop drill	Yes	81 (9.3)	66 (6.7)	4.5	0.039
	No	788 (90.7)	924 (93.3)		
Operation-based drill	Yes	91 (10.5)	75 (7.6)	4.8	0.034
	No	778 (89.5)	915 (92.4)		
Functional drill	Yes	49 (5.6)	45 (4.5)	1.2	0.283
	No	820 (94.4)	945 (95.5)		
Comprehensive drill	Yes	34 (3.9)	28 (2.8)	1.7	0.198
	No	835 (96.1)	962 (97.2)		
Emergency drill plan designing skill	Good	614 (70.7)	765 (77.3)	10.6	0.001
	Bad	255 (29.3)	225 (22.7)		
Emergency drill script writing skill	Good	431 (49.6)	538 (54.3)	4.2	0.041
	Bad	438 (50.4)	452 (45.7)		
Emergency drill form designing skill	Good	387 (44.5)	385 (38.9)	6.1	0.014
	Bad	482 (55.5)	605 (61.1)		
Emergency drill needs assessment skill	Good	279 (32.1)	394 (39.8)	11.9	0.001
	Bad	590 (67.9)	596 (60.2)		

Significant contributors from Chi-squared investigations filtered into the logistic regression equation. In the results of the binary logistic regression model, the Hausman test indicated that the model was well fitted (*p* = 0.745>0.05). The logistic regression tests revealed seven major predictors of the evaluation of the effectiveness of emergency drills. These predictors were: equipment and facilities availability, training quality, leaders’ involvement, number of training sessions, skill in emergency drill plan design, fund adequacy, and interdepartmental coordination.

The results showed that the likelihood of obtaining a lower effectiveness rating for emergency drills with insufficient equipment and facilities was 2.324 times (95% CI = 1.884–2.867) that of emergency drills with adequate equipment and facilities. Compared with participants who received high-quality training, participants who received low-quality training were 1.765 times (95% CI = 1.445–2.155) more likely to negatively evaluate the effectiveness of emergency drills. Emergency drills without leader attention were more likely to receive lower effectiveness ratings than those with leader attention (OR = 1.585). In addition, training frequency (OR = 1.539), emergency drill plan designing skill (OR = 1.494), funding guarantee (OR = 1.407), and inter-departmental coordination (OR = 1.335) were also important predictors of the effectiveness evaluation of emergency drills (Table 3)

**Table 3:** Factors influencing effectiveness evaluation of emergency drills.

Variables	Wald	*P*	OR	95% CI	
Training frequency (none) vs. (≥ 1)	17.616	<0.001	1.539	1.258	1.882
Training quality (low quality) vs. (high quality)	31.066	<0.001	1.765	1.445	2.155
No fund guarantee vs. fund guarantee	8.047	0.005	1.407	1.111	1.781
No equipment and facilities guarantee vs. equipment and facilities guarantee	62.015	<0.001	2.324	1.884	2.867
lack of leaders’ focus vs. leaders’ focus	17.174	<0.001	1.585	1.275	1.971
No inter-departmental coordination vs. inter-departmental coordination	7.480	0.006	1.335	1.085	1.641
Bad emergency drill plan designing skill vs. good emergency drill plan designing skill	11.151	<0.001	1.494	1.180	1.890

### 3.3 ISM analysis results

The specific results and the process of their analysis are described below:

1. Construct the adjacency matrix *R*: The seven significant influencing factors from the results of the binary logistic model were labeled as S1-S7. We consulted experts and conducted extensive research to establish the adjacency matrix R for the influencing factor system. We used an ISM group to analyze and discuss the matrix.

$$R=\begin{array}{c} S_{1}\\ S_{2}\\ S_{3}\\ S_{4}\\ S_{5}\\ S_{6}\\ S_{7} \end{array}\;\left[\begin{array}{ccccccc} 0 & 1 & 0 & 0 & 0 & 1 & 0\\ 0 & 0 & 0 & 0 & 1 & 0 & 0\\ 1 & 1 & 0 & 1 & 1 & 1 & 1\\ 0 & 1 & 0 & 0 & 0 & 1 & 0\\ 1 & 1 & 0 & 0 & 0 & 1 & 0\\ 0 & 1 & 0 & 0 & 0 & 0 & 0\\ 0 & 1 & 0 & 0 & 0 & 1 & 0 \end{array}\right]$$

2. Construct the reachable matrix *M*: Calculate the reachable matrix *M* from the adjacency matrix *R* of the influencing factor system.

$$M=\begin{array}{c} S_{1}\\ S_{2}\\ S_{3}\\ S_{4}\\ S_{5}\\ S_{6}\\ S_{7} \end{array}\;\left[\begin{array}{ccccccc} 1 & 1 & 0 & 0 & 0 & 1 & 0\\ 1 & 1 & 0 & 0 & 0 & 1 & 0\\ 1 & 1 & 1 & 1 & 1 & 1 & 1\\ 1 & 1 & 0 & 0 & 1 & 1 & 0\\ 1 & 1 & 0 & 0 & 0 & 1 & 0\\ 1 & 1 & 0 & 0 & 0 & 1 & 0\\ 1 & 1 & 0 & 0 & 0 & 1 & 1 \end{array}\right]$$

The set of system factors for the columns corresponding to the matrix elements with value 1 in the ith row of the reachable matrix, i.e., the reachable set *R (* $S_{i}$ *)*. The set of system factors for the row corresponding to the matrix element with value 1 in column i of the reachable matrix is the prior set *A (* $S_{i}$ *)*. The intersection of the reachable set and the prior set is the common set, i.e., *T (* $S_{i}$ *) = R (* $S_{i}$ *) ∩ A (* $S_{i}$ *)*. Accordingly, based on the reachability matrix *M*, the above set of each influencing factor is obtained as shown in the following table.

Then, by comparing the relationship between the intersection set *T (* $S_{i}$ *)* and the reachable set *R (* $S_{i}$ *)*, the first layer of the set of factors, i.e., the factors that satisfy *T (* $S_{i}$ *) = R (* $S_{i}$ *)*, is determined. Next, the stratified factors are eliminated from the reachable matrix *M* to obtain the new matrix $M_{1}$ , and the previous operation is repeated until all the factors are stratified (Table 4).

**Figure 2:**
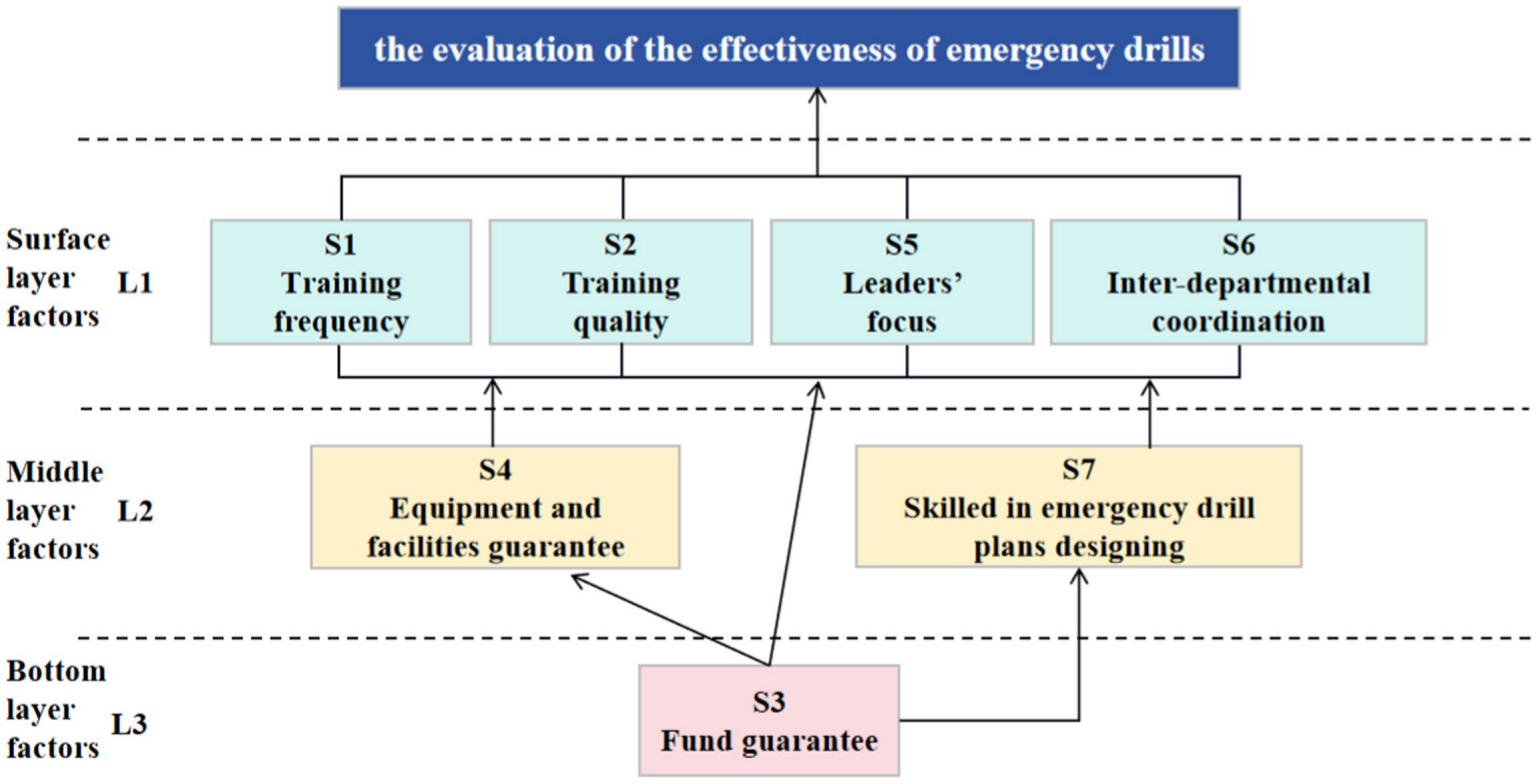
Hierarchical structure diagram of factors influencing effectiveness evaluation of emergency drills.

3. Determining the hierarchical structure *L*: Our model aims to identify the variables that influence the evaluation of the effectiveness of emergency drills for CDC personnel. We based our model on the stratification results and influence paths from the reachability matrix. We classified the system of influence factors into three levels: surface, middle, and bottom (Figure 2).

**Table 4:** Impact factor-related pooling calculations.

Factor	R (Si)	T (Si)
S1	S1、 S2、 S5、 S6	S1、 S2、 S5、 S6
S2	S1、 S2、 S5、 S6	S1、 S2、 S5、 S6
S3	S1、 S2、 S3、 S4、 S5、 S6、 S7	S3
S4	S1、 S2、 S4、 S5、 S6	S4
S5	S1、 S2、 S5、 S6	S1、 S2、 S5、 S6
S6	S1、 S2、 S5、 S6	S1、 S2、 S5、 S6
S7	S1、 S2、 S5、 S6、 S7	S7

Surface level factors. These factors have the most direct impact on the evaluation of the effectiveness of emergency drills. They include four items: training frequency (S1), training quality (S2), leaders’ focus (S5), and inter-departmental coordination (S6).

Middle level factors. These factors are the core of the system. They include two items: equipment and facilities availability (S4) and skill in emergency drill plan design (S7).

Bottom level factor. This factor is the foundation of the system that determines the overall performance. It includes one item: fund guarantee (S3).

## 4 Discussion

### 4.1 Having financial guarantee is crucial to support emergency drills

This study identified fund guarantee as the bottom-level factor that influences the evaluation of the effectiveness of emergency drills for CDC personnel. Lack of fund guarantee led to poor equipment and facilities for emergency response and ineffective emergency measures, thus compromising the evaluation of the drills.

Funding is a vital factor for conducting emergency drills in an organized and efficient manner. Without sufficient financial support, the drill may not be executed as planned. Emergency drill funding covers the expenses of materials, site rental, and personnel training needed for the drill process (25). First, equipment procurement and maintenance are essential to ensure the availability and functionality of the required tools and equipment, such as alarm systems, road signs, and rescue equipment. Adequate funds can facilitate smooth equipment procurement and maintenance for the drill process. Second, funding can be used to pay for site rental fees, environmental improvement, and temporary facilities. This ensures the site is appropriate and well-prepared. Third, personnel training and incentives are necessary for the participation and coordination of government personnel at various levels. Providing subsidies can motivate the personnel involved in the drill process.

Many developed countries assign great importance to emergency drills in the face of public health crises and have allocated significant funds toward their implementation (26). For instance, the US government has invested $21 million in drills for senior officials, highlighting the priority given to emergency preparedness (27). However, emergency drills are still in their infancy stage in China, despite substantial investments in the public health system since the SARS outbreak in 2003 (28). Therefore, it is imperative for the state and health-related financial sectors to increase their support for emergency drills. Relevant departments should include emergency drill funds in their financial budgets, establish economic evaluation and compensation mechanisms, and enhance funding mechanisms to ensure the smooth implementation of emergency drills. Moreover, it is recommended that the state establish and refine corresponding laws and regulations, prioritize emergency drills in the legal management track, and establish a comprehensive emergency drill system.

### 4.2 Adequate equipment and facilities are the basic requirements for conducting emergency drills

This study identified two intermediate-level factors that influence the evaluation of the effectiveness of emergency drills for CDC personnel: equipment and facilities availability, and skill in emergency drill plan design. The availability of adequate equipment and facilities helped the stakeholders perform their tasks confidently and comprehensively during the simulation runs, and improved their overall emergency response capabilities in areas such as communication command, emergency rescue, medical rescue, and subsequent disposal (29).

Emergency drills require various resources, such as essential personal protective equipment, mobile laboratories for pathogen detection, medical treatment facilities and equipment, proper medical waste disposal methods, and vehicles for swift mobilization in case of infectious disease outbreaks. Moreover, for catastrophic events like earthquakes, rescue and rehabilitation sites must be established. All these measures depend on an adequate supply of equipment and facilities. However, China currently faces a shortage of several categories of emergency supplies, and some of the existing emergency materials do not meet the required standards (30). Therefore, it is crucial to improve the quantity and quality of emergency stockpiles, and provide adequate facilities and equipment for emergency drills to enhance their overall performance (31).

Computer-assisted simulation technology can help address these issues (6, 32). In China, the “13th Five-Year Plan for the Construction of a National Emergency Response System” advocates for the creation of a national public safety emergency experience base. The utilization of virtual simulation technology to replicate various disaster scenes, characterized by minimal space requirements, reduced costs, and reusability, has a vast potential in the emergency management domain (33). In this modern technological era, cost-effective emergency drills would be challenging to implement without the support of advanced electronic technologies such as 3D simulation systems or VR systems (34).

### 4.3 A qualified design of emergency drill plan is the key

The success of emergency drills depends largely on the design of the emergency drill plan, which serves as a blueprint for conducting the drills. Therefore, meticulous planning is essential, as it directly affects the drill’s impact and management (35, 36). A well-designed emergency drill plan can enhance the leadership’s ability to prioritize and coordinate the response efforts, and improve the flow and sequence of the processes for successful deployment. Hence, creating hypothetical scenarios requires a comprehensive and thoughtful approach.

Fundamentally, drills are about “answering questions.” Every scenario within the drill atmosphere requires a response, and if these “scenarios” are viewed as “questions,” then responding to the scenarios equates to “answering questions (10).” The scope of “problems” is broad, encompassing all situations related to activating emergency responses before, during, and after an incident. When the scenario construction and drill scene settings are rigorous, every sentence, phrase, word group, and even punctuation in the scenario description can be a “problem” that necessitates a thoughtful answer (10, 37, 38). All of this relies on the meticulous advance design of the emergency drill plan. However, at present, there are significant shortcomings in the overall design of emergency drill plans. Many designs are too ordinary and disregard the unexpected nature of public health emergencies. This makes the emergency drills lack authenticity and a scientific basis.

To address this issue, the design of the rehearsal plot should be as realistic and diverse as possible to ensure the quality of emergency drills. Additionally, in the planning of drills, it is possible to deconstruct the functions of contingency plans according to certain standards, breaking them down to the smallest executable units. This involves specific emergency functions such as the roles and responsibilities outlined in the plan, emergency mobilization, resource management, facility and equipment support, early warning and announcements, traffic control, management of evacuated personnel, medical services, external reinforcement, situation control and on-site recovery, documentation and investigation (10). The aim is to achieve the exercise objectives for each module as much as possible.

### 4.4 Establish multi-sectorial coordination mechanism and strengthen the leadership’s attention

This study showed that the evaluation of the effectiveness of emergency drills is directly influenced by the surface level factors, which include four items: training frequency, training quality, inter-departmental coordination, and leaders’ focus. Some units did not organize or conduct the drills effectively or regularly, resulting in low attention and participation from the personnel. This made it difficult to identify and solve problems, exercise the team, and enhance the overall capabilities, thus reducing the evaluation of the effectiveness of emergency drills (28). Moreover, poor training quality prevented the full realization of the functions of the drills.

Notably, insufficient leaders’ involvement led to superficial drills with low-quality implementation. In China, the government budget allocation authority is primarily controlled by the central, provincial, municipal, and county-level financial departments. Due to decades of decentralization reforms, local governments are responsible for financing local health and other social services. Although it is a national policy that requires local CDCs to undergo emergency training, only wealthier provinces and municipalities with more funds and resources, where leaders prioritize it, provide sufficient support for emergency drills. Compared to traditional knowledge-based training methods such as lectures, courses, or workshops - often funded by the central government as a nationwide earmarked program - skill-based emergency drills rarely receive funds from the center. Therefore, the financing responsibility mainly falls on the shoulders of local governments, implying the need for extra human, material, and financial resources. If leaders in poor areas fail to recognize the importance and irreplaceability of emergency drills and consider them a waste of resources, they may be reluctant to allocate extra funds. To improve this situation, various methods such as financial support, technology transfer, leadership training, and legal enforcement should be adopted.

Finally, departmental coordination was a vital aspect of the drills. Without close collaboration between departments, it was hard to achieve rapid response and synergy. However, currently, emergency drills mainly consist of individual departmental activities, with administrative units operating independently and lacking a multi-agency coordination mechanism (39). The absence of coordination, coupled with conflicts among agencies, can often result in extensive time loss, resource waste, duplication, and uncoordinated and inappropriate responses (40). Therefore, to improve effectiveness, teams should undergo comprehensive training to develop knowledge, skills, and attitudes; enhance coordination; foster a shared mental model and accurate expectations of team requirements; and encourage adaptability and flexibility (41). Multi-agency emergency drills can establish and strengthen relationships, bring together individuals from different backgrounds to work as a team, clarify goals, understand roles and responsibilities, and appreciate each agency’s strengths and weaknesses (42, 43).

### 4.5 Limitations of this study

Although this study used the Logistic-ISM method to identify the factors related to the CDC staff’s evaluation of the emergency drill outcomes, and further derived the hierarchical structure of the related factors, some limitations still need to be addressed.

First, this survey may not capture all the potential factors that may cause reasoning bias, and the presence of omitted variables remains an area for future research. Second, this study used cross-sectional data for analysis, which cannot obtain causal effects. Future studies can use longitudinal data sets to capture more accurate relationships between the research variables and the CDC staff’s evaluation of the emergency drill outcomes. Third, this study only surveyed the data from Heilongjiang Province, which limits the generalizability of the research results. Future studies can expand the sample size, include more provinces in China, and explore the potential differences in the results obtained in different regions. Finally, this study’s sampling design used a multistage cluster sampling method, but did not account for this in the statistical analysis, which could affect the accuracy of parameter estimation and hypothesis testing. Future research should use more appropriate statistical methods to deal with the multistage sampling issue.

## 5 Conclusion

This paper explores the factors that affect the evaluation of the effectiveness of emergency drills and their hierarchical structure based on actual research data. The paper uses Logistic model and ISM analysis to identify and analyze the factors. The results of the study show that the evaluation of the effectiveness of emergency drills is the result of a combination of factors. Firstly, seven factors have significant influence on the effectiveness of emergency drills. They are equipment and facilities guarantee, training effectiveness, leaders’ focus, training frequency, skill in emergency drill plans design, fund guarantee, and inter-departmental coordination. Secondly, the seven dominant factors are both independent and interrelated, and are at three different levels. Among them, training frequency, training quality, leaders’ focus, and coordination between departments are factors that directly affect the effectiveness of emergency drills for public health emergencies; they are the surface influences. The intermediate influences are equipment and facilities guarantee and skill in emergency drill plans design. Fund guarantee is the deep-rooted factor.

## Author contributions

RZ: Data curation, Formal analysis, Investigation, Methodology, Software, Writing – original draft. AR: Writing – original draft, Writing – review & editing. NC: Formal analysis, Writing – review & editing. XW: Formal analysis, Writing – review & editing. XQ: Formal analysis, Writing – review & editing. QKW: Writing – review & editing, Investigation, Writing – original draft. YW: Writing – review & editing, Investigation, Writing – original draft. ZK: Formal analysis, Supervision, Writing – review & editing. JL: Formal analysis, Funding acquisition, Methodology, Resources, Supervision, Validation, Writing – review & editing. QHW: Formal analysis, Methodology, Visualization, Writing – original draft.

## Data availability statement

The original contributions presented in the study are included in the article/supplementary material, further inquiries can be directed to the corresponding author/s.

## Ethics statement

The studies involving humans were approved by the Ethics Committee of Harbin Medical University approved the study. The studies were conducted in accordance with the local legislation and institutional requirements. The participants provided their written informed consent to participate in this study.

## References

[1] Gebbie KM Valas J Merrill J Morse S. Role of exercises and drills in the evaluation of public health in emergency response. Prehosp Disaster Med (2006) 21:173–82. doi: 10.1017/s1049023x00003642, PMID: 16892882

[2] Ablah E Konda KS Konda K Melbourne M Ingoglia JN Gebbie KM. Emergency preparedness training and response among community health centers and local health departments: results from a multi-state survey. J Community Health (2010) 35:285–93. doi: 10.1007/s10900-010-9236-7, PMID: 20379843

[3] Hites LS Sass MM D’Ambrosio L Brown LM Wendelboe AM Peters KE. The preparedness and emergency response learning centers: advancing standardized evaluation of public health preparedness and response trainings. J Public Health Manag Pract (2014) 20 Suppl 5:S17–23. doi: 10.1097/PHH.0000000000000066, PMID: 25072484

[4] Dausey DJ Buehler JW Lurie N. Designing and conducting tabletop exercises to assess public health preparedness for manmade and naturally occurring biological threats. BMC Public Health (2007) 7:92. doi: 10.1186/1471-2458-7-92, PMID: 17535426 PMC1894789

[5] Davis M Reeve M Altevogt BM. Nationwide response issues after an improvised nuclear device attack: Medical and public health considerations for neighboring jurisdictions: Workshop summary. Washington, D.C, United States: National Academies Press (2013). 24199263

[6] Andreatta PB Maslowski E Petty S Shim W Marsh M Hall T. Virtual reality triage training provides a viable solution for disaster-preparedness. Acad Emerg Med (2010) 17:870–6. doi: 10.1111/j.1553-2712.2010.00728.x, PMID: 20670325

[7] Agboola F Bernard D Savoia E Biddinger PD. Development of an online toolkit for measuring performance in health emergency response exercises. Prehosp Disaster Med (2015) 30:503–8. doi: 10.1017/s1049023x15005117, PMID: 26369757

[8] Chinese Center for Disease Control and Prevention. Notice on the issuance of “Health Emergency Drill Technical Guidelines (2013 Edition)” by the Chinese Center for Disease Control and Prevention. (2013). Available at: https://m.chinacdc.cn/xwzx/tzgg/201312/t20131231_92083.html.

[9] Boslough M Jennings B Carvey B Fogleman W. FEMA asteroid impact tabletop exercise simulations. Procedia Engineering (2015) 103:43–51. doi: 10.1016/j.proeng.2015.04.007

[10] Zhang XB. Some basic issues on emergency drills. J Henan Polytech Univ (2019) 20:54–9.

[11] Hou SK Lv Q Ding H Zhang YZ Yu BG Liu ZQ. Disaster medicine in China: present and future. Disaster Med Public Health Prep (2018) 12:157–65. doi: 10.1017/dmp.2016.71, PMID: 27349809

[12] Biddinger PD Savoia E Massin-Short SB Preston J Stoto MA. Public health emergency preparedness exercises: lessons learned. Public Health Rep (2010) 125:100–6. doi: 10.1177/00333549101250s514, PMID: 21133066 PMC2966651

[13] Lurie N Wasserman J Nelson CD. Public health preparedness: evolution or revolution? Health Aff (2006) 25:935–45. doi: 10.1377/hlthaff.25.4.935, PMID: 16835172

[14] Yousefi M Ferreira RPM. An agent-based simulation combined with group decision-making technique for improving the performance of an emergency department. Braz J Med Biol Res (2017) 50:e5955. doi: 10.1590/1414-431x20175955, PMID: 28380196 PMC5423739

[15] Eastwood K Durrheim D Merritt T Massey PD Huppatz C Dalton C. Field exercises are useful for improving public health emergency responses. Western Pac Surveill Response J (2010) 1:12–8. doi: 10.5365/wpsar.2010.1.1.003, PMID: 23908875 PMC3729048

[16] Chandrasekhar CP Ghosh J. Information and communication technologies and health in low income countries: the potential and the constraints. Bull World Health Organ (2001) 79:850–5. doi: 10.1590/S0042-96862001000900010, PMID: 11584733 PMC2566653

[17] Wang Z Liang WN Di ZQ. Discussion on the content and methods of health emergency training in disease control institutions. Chin J Public Health (2009) 25:408–9.

[18] Zhong S Clark M Hou XY Zang YL Fitzgerald G. Development of hospital disaster resilience: conceptual framework and potential measurement. Emerg Med J (2014) 31:930–8. doi: 10.1136/emermed-2012-202282, PMID: 24028975

[19] Liu Y. China’s public health-care system: facing the challenges. Bull World Health Organ (2004) 82:532–8. doi: 10.1590/S0042-96862004000700011 15500285 PMC2622899

[20] Ouyang Y. Earthquake tests China’s emergency system. Lancet (2013) 381:1801–2. doi: 10.1016/S0140-6736(13)61105-8, PMID: 23717832 PMC7138371

[21] You C Chen X Yao L. How China responded to the may 2008 earthquake during the emergency and rescue period. J Public Health Policy (2009) 30:379–94. doi: 10.2307/40542233, PMID: 20029427

[22] Ning N Wu QH Shang JW. Analysis on the current situation of emergency drills in Heilongjiang province disease prevention and control institutions. *Chinese healthcare*. Management (2010) 27:351–3.

[23] Xu Y Liu H Lyu J Xue Y. What influences farmers’ adoption of soil testing and formulated fertilization Technology in Black Soil Areas? An empirical analysis based on logistic-ISM model. Int J Environ Res Public Health (2022) 19:15682. doi: 10.3390/ijerph192315682, PMID: 36497757 PMC9738926

[24] Li X Zhang H Wang M. Analysis of factors influencing the decision-making behavior of beef cattle farmers: an empirical analysis based on logit-ISM model. Animals (2022) 12:3470. doi: 10.3390/ani12243470, PMID: 36552390 PMC9774255

[25] Ning N Wu QH Hao YH Shang JW. Factor analysis of influencing factors in emergency drills. Chin J Health Res (2013) 16:18–32.

[26] Hong K Chen QH. Development and inspiration of American emergency drill system. *China emergency*. Management (2011) 9:54–9.

[27] Guo H Zhao W Pan C Qiu G Xu S Liu S. Study on the influencing factors of farmers’ adoption of conservation tillage Technology in Black Soil Region in China: a logistic-ISM model approach. Int J Environ Res Public Health (2022) 19:7762. doi: 10.3390/ijerph19137762, PMID: 35805419 PMC9266123

[28] Kang Z Wu QH Gao LJ Ning N Shang JW Jiao ML. Analysis on influencing factors of satisfaction in disease prevention and control institutions' emergency drills based on logistic regression analysis. Chin J Health Res (2013) 16:25–7.

[29] Li YG Liu SB. Practice and reflection on health supervision emergency drills. Chin J Health Sup (2014) 21:498–500.

[30] Drury J Cocking C Reicher S Burton A Schofield D Hardwick A. Cooperation versus competition in a mass emergency evacuation: a new laboratory simulation and a new theoretical model. Behav Res Methods (2009) 41:957–70. doi: 10.3758/BRM.41.3.95, PMID: 19587213

[31] Pourhosseini SS Ardalan A Mehrolhassani MH. Key aspects of providing healthcare Services in Disaster Response Stage. Iran J Public Health (2015) 44:111–8. PMID: 26060782 PMC4449997

[32] Kwegyir-Afful E Hassan TO Kantola JI. Simulation-based assessments of fire emergency preparedness and response in virtual reality. Int J Occup Saf Ergon (2022) 28:1316–30. doi: 10.1080/10803548.2021.1891395, PMID: 33591217

[33] Zhang XB Zhang R Xie YB. Review on the management of emergency drills in China. J Saf Sci Technol (2016) 12:68–73.

[34] Kinateder M Müller M Jost M Mühlberger A Pauli P. Social influence in a virtual tunnel fire--influence of conflicting information on evacuation behavior. Appl Ergon (2014) 45:1649–59. doi: 10.1016/j.apergo.2014.05.014 24947002

[35] Ebbeling LG Goralnick E Bivens MJ Femino M Berube CG Sears B. A comparison of command center activations versus disaster drills at three institutions from 2013 to 2015. Am J Disaster Med (2016) 11:33–42. doi: 10.5055/ajdm.2016.0222, PMID: 27649749

[36] Burke RV Lehman-Huskamp K Whitney RE Arora G Park DB Mar P. Checklist use in evaluating pediatric disaster training. Am J Disaster Med (2015) 10:285–94. doi: 10.5055/ajdm.2015.0210, PMID: 27149309

[37] Xie YB Zhang XB. Discussion on the "task-responsibility-performance" model in major road traffic accident rescue. J Saf. Sci. Technol. (2015) 11:18–25.

[38] Wang CP Yan LP Wang J. Exploratory practice and reflection on a command center's emergency drill. *China emergency*. Management (2014) 5:16–21.

[39] Milis G Kolios P Van Melick G Staykova T Helsloot I Ellinas G. Integrated modelling of medical emergency response process for improved coordination and decision support. Healthc Technol Lett (2016) 3:197–204. doi: 10.1049/htl.2016.0039, PMID: 27733927 PMC5048341

[40] Kako M Arbon P Mitani S. Disaster health after the 2011 great East Japan earthquake. Prehosp Disaster Med (2014) 29:54–9. doi: 10.1017/S1049023X14000028 24451332

[41] Kanadanian KV Haan CK. Postcrisis redevelopment of sustainable healthcare systems. Am J Disaster Med (2014) 9:247–58. doi: 10.5055/ajdm.2014.0177, PMID: 25672328

[42] Crichton M Kelly T. Developing emergency exercises for hazardous material transportation: process, documents and templates. J Bus Contin Emer Plan (2012) 6:32–46. PMID: 22948104

[43] Gresham L Ramlawi A Briski J Richardson M Taylor T. Trust across borders: responding to 2009 H1N1 influenza in the Middle East. Biosecur Bioterror (2009) 7:399–404. doi: 10.1089/bsp.2009.0034, PMID: 20028248

